# Milk Product Safety and Household Food Hygiene Influence Bacterial Contamination of Infant Food in Peri-Urban Kenya

**DOI:** 10.3389/fpubh.2021.772892

**Published:** 2022-02-08

**Authors:** Vivian Hoffmann, Sheillah Simiyu, Daniel K. Sewell, Kevin Tsai, Oliver Cumming, Jane Mumma, Kelly K. Baker

**Affiliations:** ^1^Markets Trade and Institutions Division, International Food Policy Research Institute, Washington, DC, United States; ^2^Africa Population Health Research Center, Nairobi, Kenya; ^3^Department of Biostatistics, University of Iowa, Iowa City, IA, United States; ^4^Department of Occupational and Environmental Health, University of Iowa, Iowa City, IA, United States; ^5^Department of Disease Control, London School of Hygiene and Tropical Medicine, London, United Kingdom; ^6^Department of Community Nutrition, Great Lakes University of Kisumu, Kisumu, Kenya

**Keywords:** infant food, food systems, household hygiene, food exposure, milk contaminants, foodborne bacterial pathogens, food treatment and sterilization, enteric diseases

## Abstract

**Background:**

Milk is a common infant food in peri-urban Kenya that can transmit diarrhea-causing enteric pathogens. Little is known about how contamination of milk at point of purchase and household handling of milk-based infant foods contribute to infant exposure to enteric pathogens.

**Objective:**

To compare the prevalence and concentrations of bacterial indicator organisms and enteric pathogens in unpackaged, fresh pasteurized, and ultra-high temperature (UHT) treated milk at purchase and assess the influence of the type of milk used to prepare infant food on contamination of this food.

**Methods:**

Paired samples of purchased milk and infant food prepared with this milk were obtained from 188 households in low-income neighborhoods in Kisumu, Kenya. Samples were cultured on selective media to isolate S*almonella enterica, Shigella* spp., *Klebsiella aerogenes, Proteus* spp., and *Escherichia coli*, with pathogens validated by PCR. Probability of detection of these bacteria was compared by milk product treatment and packaging method, and between milk at point of purchase vs. food at point of infant consumption.

**Results:**

Unpackaged milk was most contaminated at point of purchase, but bacterial contamination was also present in pasteurized and UHT milk at purchase. Presence of bacteria in UHT and fresh pasteurized milk at purchase predicted presence of the same bacteria type in infant food. Prevalence of bacterial contamination and concentration level for bacterial indicators generally increased between point of purchase and consumption in UHT and fresh pasteurized milk-based food but decreased in unpackaged milk-based food. Prevalence of the four fecal bacteria were similar in infant foods prepared with each type of milk.

**Conclusion:**

Both pre-market contamination and post-purchase handling influence the likelihood of infants ingesting foods contaminated by diarrheal pathogens.

## Introduction

Morbidity and mortality among children under 5 years of age accounts for 40% of the global burden of foodborne disease (1). Contaminated food is a major pathway of bacterial exposure among infants (2, 3), and there is evidence that food consumed by infants is more contaminated than foods consumed by adults (4). Milk may be particularly high risk as it frequently contains a higher number of fecal coliforms than many other foods consumed by infants (2, 5, 6). Recent research in Kenya, where cow's milk is an important infant food, shows that milk stored in the household for feeding to young children is more likely to be contaminated with an enteric pathogen than other weaning foods (7). An important question for addressing the problem of unsafe infant foods is the source of this contamination: Does milk already contain pathogens when it is brought into the household, or do these enter through unhygienic household food preparation, treatment, and storage conditions?

Kenya's dairy value chain is complex, with a variety of rural, peri-urban, and urban independent farms of small to large size that sell milk in bulk through dairy cooperatives to formal milk processing plants (8). These plants package milk for sale as fresh pasteurized (refrigeration required) and Ultra High Temperature treated (UHT) “Long Life” milk (shelf stable). Farms may also sell milk to road-side or mobile vendors who sell unpackaged milk directly to consumers, meaning vendors fill bags or other containers from bulk vessels. This unpackaged milk may be either raw or pre-boiled. Previous quantitative risk assessments of disease transmission through milk in Kenya have focused on informally marketed raw milk. These have used self-reported boiling behavior to estimate the proportion of households who boil milk prior to consumption and have assumed that boiling is 100% effective against *Escherichia coli* O157:H7 and *Cryptosporidium* spp. (9, 10). As the vast majority of households typically report boiling milk purchased from the informal market prior to consumption, previously estimated risks of exposure to foodborne bacteria through milk consumption have been small.

However, these previous studies do not consider several important factors. First, consumption of pasteurized milk is high among Kenya's rapidly growing urban population. Nationally representative data collected in 2015 show that 66% of households in Nairobi, Kenya's largest city, and 60% of those in peri-urban Kisumu, the site of the present study, had consumed either fresh pasteurized or UHT milk over the past 7 days (11). An earlier study found that while consumption of pasteurized milk in Nairobi increased with income, even households in the lowest income quintile were as likely to consume pasteurized as raw milk (12). Due to the perception that it is ready to drink, packaged milk may not be boiled, even by those who typically boil unpackaged, informally marketed milk. While this perception is likely to be true, surveillance evidence verifying that packaged milk consistently meets the East African Community (EAC) standard is lacking.

Second, processed milk may be safer than raw milk, but still contain pathogens. Some pathogens, such as heat-resistant spores of *C. botulinum* or *B. cereus*, can survive pasteurization (heated to 71–74°C for 15–40 seconds (s)) (13). The UHT treatment of milk [135–140°C for 6–10 seconds (s)] more efficiently destroys vegetative pathogens and heat-resistant spore forming pathogens, allowing this milk to be stored at room temperature for longer periods of time, but is still not full sterilization. Further, processed milk may be contaminated with pathogens after treatment. This may occur if processing equipment is not properly cleaned, if packaging materials are contaminated, or if the very low levels of pathogens remaining in milk post-pasteurization are able to multiply due to failures in the cold chain. *Listeria monocytogenes* from biofilms on processing equipment has caused foodborne outbreaks in several settings (14).

Third, survey respondents may misreport boiling behavior to researchers due to a desire to be seen to be doing the “right thing.” Finally, even if households are boiling milk prior to consumption, re-contamination of the boiled milk may occur through utensils and hands that have come into contact with unboiled milk, household surfaces, or airborne dust. If contamination is bacterial and milk is stored at room temperature, contamination levels can rise over time with bacterial replication. As a single batch of prepared infant food is often consumed over multiple feeding events spanning several hours, the potential for bacterial replication during storage is high.

The aim of this study is to assess food safety risks associated with different types of purchased milk given to infants at 8 months of age in Kenya, where diarrheal disease mortality is high (15). Specifically, we assess the contributions of microbial contamination at time of purchase, and contamination introduced during handling within the household, to the overall risk of contamination of milk-based infant food.

## Materials and Methods

### Study Design

**Figure 1:**
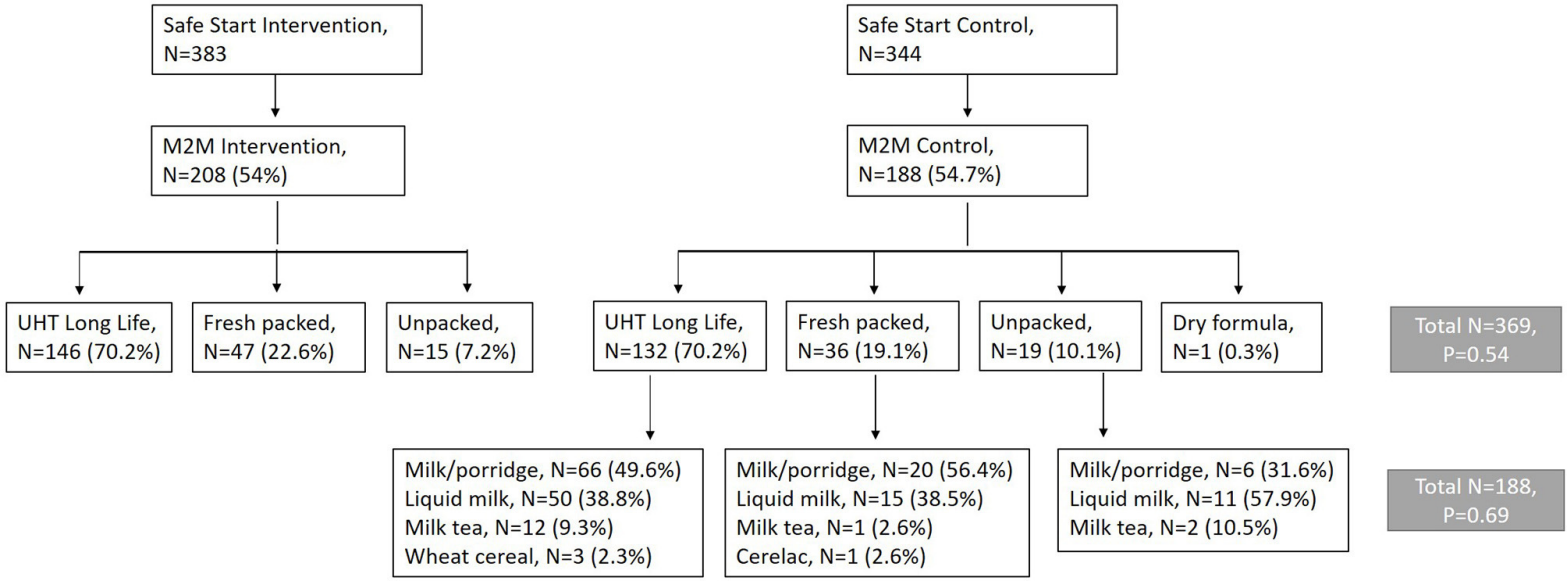
Flow diagram showing recruitment of 396 Market to Mouth households from 726 caregivers enrolled in the Safe Start Study who completed a mid-intervention survey and purchased milk for feeding their infant and submitted a food sample for microbiological testing.

The Market to Mouth study is a separately funded study embedded within a cluster-randomized randomized controlled trial called Safe Start, for the purpose of better understanding the mechanisms by which the Safe Start intervention influenced infant food safety. Both studies were based in the Nyalenda A and Nyalenda B wards of peri-urban Kisumu. These densely populated wards are characterized by lack of improved sanitation facilities, use of county-provided water points, poor housing, and high rates of poverty. The Safe Start study evaluates the effect of a food hygiene intervention targeting early childhood exposure to enteric pathogens through contaminated food (ClinicalTrials.gov ID: NCT03468114) (16). The protocol includes a midline visit at 8 months of age to observe the caregiver prepare food and feed the child, and to take a food sample for microbial testing. The Market to Mouth study uses food sample data collected at this midline visit, and paired samples purchased from the market vendors where caregivers procured milk fed to the infants tracked by Safe Start, to examine potential pathogen transmission patterns. In this manuscript, we analyze 396 milk samples purchased directly from vendors patronized by all (intervention and control) Safe Start households, and paired infant food samples from the 188 households among these who were assigned to the Safe Start control group (Figure 1). Practices by caregivers in the control group are expected to reflect those of this population in the absence of a food safety intervention.

### Human Subjects Research

Approval for the collection of infant food samples was obtained from Great Lakes University of Kisumu (Ref: GREC/010/248/2016), London School of Hygiene and Tropical Medicine (Ref: 14695), and the University of Iowa (Ref: 00000099) (16). An informed consent form was read to caregivers in their preferred language. If caregivers consented to participate in the study, they signed consent forms in the presence of a community witness and were given copies of the informed consent form for their records. Participants were allowed to withdraw at any time.

### Patient and Public Involvement

Formative research on caregiver experiences and infant food safety (7, 17), and the challenges for Community Health Volunteers (CHVs) in delivery of health care information (18) informed our study design. The design of the Safe Start intervention was optimized through an interactive pilot study (19) and CHVs were involved in the implementation of the study (16). Community knowledge dissemination meetings were convened after the study to discuss results with the community.

### Data Collection and Food Sampling

When scheduling the Safe Start midline visit, the research team inquired about what the caregiver intended to prepare for the child to eat the following day. If the caregiver planned to feed the child milk or food prepared with milk, the team arranged with the caregiver for a study enumerator to meet and travel with the caregiver to procure the milk, whether this was done the same evening, the following morning, or just before the food was prepared. Only caregivers who fed their infants milk or food made with milk, for example milk tea or milk porridge, are included in the present study (54% of 733 Safe Start caregivers). The enumerator accompanying the caregiver during milk purchase recorded the type of milk (unpackaged, fresh pasteurized, UHT, or powdered infant formula), price paid, volume obtained, and whether the milk was refrigerated at the point of purchase. The enumerator then purchased an additional unit of the same milk product (from the same vessel, if the milk was unpackaged, or the same brand, if the milk was fresh packaged or UHT) from the vendor for laboratory analysis.

During the Safe Start midline visit, which typically occurred in the morning, the Safe Start research team observed the caregiver's food preparation and infant feeding practices and collected a food sample for laboratory analysis. In addition to the morning visit, the Safe Start team scheduled a time in the afternoon, per convenience of the caregiver, to collect another sample of the infant food that had been prepared in the morning if it was still being used by that time. The afternoon visit was scheduled as late as possible, to capture the full influence of household storage practices on the microbial quality of infant food. Afternoon food samples were obtained from 163 of the 188 households from which infant food was collected. All assays were conducted on the afternoon food sample if one was obtained, and on the morning sample otherwise.

Caregivers were asked to provide a spoonful of solids (~5 grams) or ~ 2 fluid ounces of this food to minimize unnecessary oversampling of food that would otherwise be fed to the infant, using the same utensil as they were currently using to feed the infant. Both vendor and household samples were barcode labeled to match key identifiers of the household ID. All samples of milk at purchase and infant food were transported to the laboratory on ice packs in a cooler within 4 hours (h) of collection.

### Laboratory Methods and Comparison of Milk Against Regulatory Standard

Samples were processed within 2 h of receipt at the laboratory by Alkaline Phosphatase (ALP) assays to assess pasteurization status, by bacterial pre-enrichment and culture assays for select foodborne pathogens and microbial indicators, and direct DNA and RNA extraction for quantitative molecular analysis for a broad array of enteric pathogens and human and bovine microbial source tracking markers.

#### Pasteurization Assay

ALP assays (Charm Sciences, Inc., Lawrence, MA) were used to determine whether milk or foods containing milk had been sufficiently boiled at either high enough temperatures or long enough time periods to achieve pasteurization conditions. Prior to analyzing any food samples, technicians checked outcomes of positive and negative controls. If the device did not correctly read these controls, food samples were not analyzed that day.

#### Analysis of Enterobacteriaceae spp. Contamination

Most bacteria can survive and grow in cold temperatures, but that temperatures of <8–10°C suppress growth rate such that concentration might at most double during an overnight incubation at 3–5°C, as opposed to a 50-fold or more increase in concentration in the same time period at 30°C or greater (20, 21). We take advantage of this bacterial property to recover heat-injured or metabolically inert viable bacteria in infant food, while slowing the growth rate to quantify bacterial concentrations at the time of sampling. A 2 ml sample of liquid food or 300 mg of solid food was mixed with 2 ml of a Peptone Enrichment Broth and incubated at 3–5°C for 24 h (22). The next day, samples were incubated at 41°C for 1 h to trigger bacterial replication metabolism. Microbial presence and concentration in food samples were determined using culture-based isolation and phenotyping of a subset of bacteria commonly found in the digestive tracts and feces of humans and cattle, and common foodborne pathogens: *Salmonella* spp. (including *S. enterica*), *Shigella sonnei* (*S. sonnei*), *Enterobacter aerogenes*, generic *E. coli* including pathogenic EHEC 0157, and *Proteus* spp. After pre-enrichment, 1 ml, 100 μl, and 10 μl serial dilutions volumes of sample were vacuum filtered through 0.45 μM membrane filters (Millipore, MA, USA) and cultured on the selective and differential chromogenic medium, *E. coli* O157: H7 MUG agar (Sigma-Aldrich, #44782, St. Louis, MO, USA) for 24 h at 35–37°C. Presumptive bacterial pathogen presence was determined by counting individual colony forming units (cfu) for each phenotype, according to the manufacturer's protocol. One negative environmental control was performed on each day of processing. Commercially available bacterial reference strains fof each target organism were acquired from BEI Resources (Manassas, VA, USA) for use as positive controls to verify performance of each batch of pre-enrichment media or media plates, and to confirm pathogen identity during PCR.

#### Validation of Pathogen Phenotype

Five colonies of each phenotype were picked into 100 μl of molecular grade water and boiled at 100°C for 5 min to destroy bacteria cell structure and release its DNA, followed by centrifuging at 12,000 g for 5 min to precipitate cell debris and obtain clean supernatant. DNA were frozen at −20°C until a PCR test could be performed to verify strain type. A qualitative polymerase chain reaction (PCR) assay was used to determine whether presumptive *Salmonella* spp. colonies carried the *ttr* gene indicative of *S. enterica*, presumptive EHEC 0157 colonies carried the *rdbE*, and presumptive *S. sonnei* carried *virG* and *ipaH* virulence genes. PCR template included 2 μl of the DNA template, 10 μl of the Taq master mix, 1.6 μl each of 5 μM of forward and reverse primer **(**gene target: *rbdE* for *E. Coli* O157: H7, *virG* and *ipaH* for *Shigella* spp., and *ttr* for *S. enterica***)**, and 4.8 μl of nucleic acid-free water. PCR was performed in an Eppendorf thermocycler (Eppendorf, Germany) under cycling conditions: 94°C for 3 min, followed by 40 cycles of 94°C for 30 s, 60°C for 1 min, and 72°C for 1 min, then finished at 72°C for 10 min. The amplified samples underwent gel electrophoresis to confirm amplicon presence. Water controls were used during PCR and gel electrophoresis to detect issues with background contamination. Failure to detect these genes meant samples were classified as negative for *S. enterica*, EHEC 0157, and *S. sonnei*, respectively.

#### Comparison Against Regulatory Standard

To assess whether milk samples met the local guidelines for microbial contamination, we compare the *E. aerogenes* plate count in processed and raw milk against the East African Community (EAC) milk standard. This standard describes maximum acceptable coliform bacteria plate counts for pasteurized (10 cfu/ml) and raw (50,000 cfu/ml) milk (23, 24). The total coliform plate count could be higher than the *E. aerogenes* plate count, so this provides a lower bound of true non-compliance with this element of the EAC standard. We compare infant food samples against the EAC pasteurized milk standard.

### Statistical Analysis

Statistical analysis was performed using Stata version 16.0 (StataCorp, 2019). We omit the single observation of powdered infant formula from the statistical analysis. The analysis sample thus consists of 395 vendor and 187 fluid milk samples. Analysis of statistical power is provided in Supplementary Tables S1–S3 and associated text within the Supplementary Materials.

We use Fisher's exact test to compare the microbial prevalence across milk types (unpackaged, fresh pasteurized, and UHT), across brands at purchase, and across infant foods by type of milk used in preparation. A likelihood ratio test based on a negative binomial regression model with milk type indicators is used to compare microbial diversity across milk types. As a robustness test of the comparisons across milk types, we estimate marginal effects of vendor milk type on microbial presence and diversity, controlling for modifiers (refrigeration status for vendor milk and food type for infant food samples), using logistic and negative binomial regressions, respectively.

When comparing paired data (vendor samples and paired infant food samples), we use McNemar's exact test for binary indicators of microbial presence, and a negative binomial generalized linear model (GLM) with correlated standard errors within paired samples for microbial diversity.

We calculate the odds ratio for detection of each organism in infant food based on whether the same organism was found in the milk used to prepare it.

To characterize changes in microbial concentration between vendor milk and infant food samples, we first exclude observations for which a given organism is detected in neither the vendor milk nor the infant food sample, or for which microbial concentration at the maximum limit of analysis is observed in both samples. We then assess whether the proportion of paired samples in which microbial concentration increased vs. decreased between purchase and child feeding is influenced by vendor milk type using Fisher's exact test.

### Data Access

A curated dataset with contamination data for milk sources can be provided immediately upon request. The data on matched infant food contamination is a secondary outcome of the Safe Start clinical trial (16) and can be made publicly accessible as a de-identified dataset after trial results have been reported.

## Results

### Sources of Milk and Types of Milk Used for Infant Food

Of the 396 caregiver milk purchases observed, 90.4% were from small shops known as dukas, 5.3% from milk bars, 0.5% from roadside vendors, 1.0% were obtained from study households' own cows, 1.8% from neighbors' cows, and 0.5% from larger shops (Supplementary Table S4). The most common type of milk purchased for infant feeding was long-life UHT milk, at 70% of all samples, followed by fresh pasteurized milk at 21%, and unpackaged milk at 8.6%. Powdered infant formula was purchased by a single caregiver. Almost all pasteurized and UHT milk and the single observation of infant formula were purchased from dukas. Most of the unpackaged milk was purchased from milk bars (21 purchases), while two caregivers purchased such milk from a roadside vendor, and the remainder (11 purchases) obtained unpackaged milk from a neighbor or the household's own cow.

### Vendor Practices and Milk Contamination at Point of Purchase

Among milk types that require refrigeration (non-UHT), 77% of the 82 samples purchased from dukas were refrigerated and 76% of the 21 purchased from milk bars were refrigerated (Supplementary Table S4). The two roadside vendors and neighbors from whom milk was purchased informally did not refrigerate the milk offered for sale. The single larger shop in the sample from which fresh pasteurized milk was purchased also failed to refrigerate. Most vendors of unpackaged milk stored it in wide-mouthed containers with lids (59% = 20/34), 15% kept it in containers ready for sale, and 21% stored milk in wide-mouthed containers without lids, which could allow flies to enter.

We were only able to obtain data on ALP inactivation for 37 milk samples due to unreliability of the Charm device used for testing (positive and negative controls inconsistent). ALP assays of 36 packaged milk samples (fresh packaged or UHT) indicated that most of this milk was heated to pasteurization temperatures sufficient to kill most bacteria. One UHT milk sample still had active ALP enzyme activity suggesting insufficient treatment. Only one unpackaged milk sample was tested, and the result indicated insufficient or absent pasteurization.

**Figure 2:**
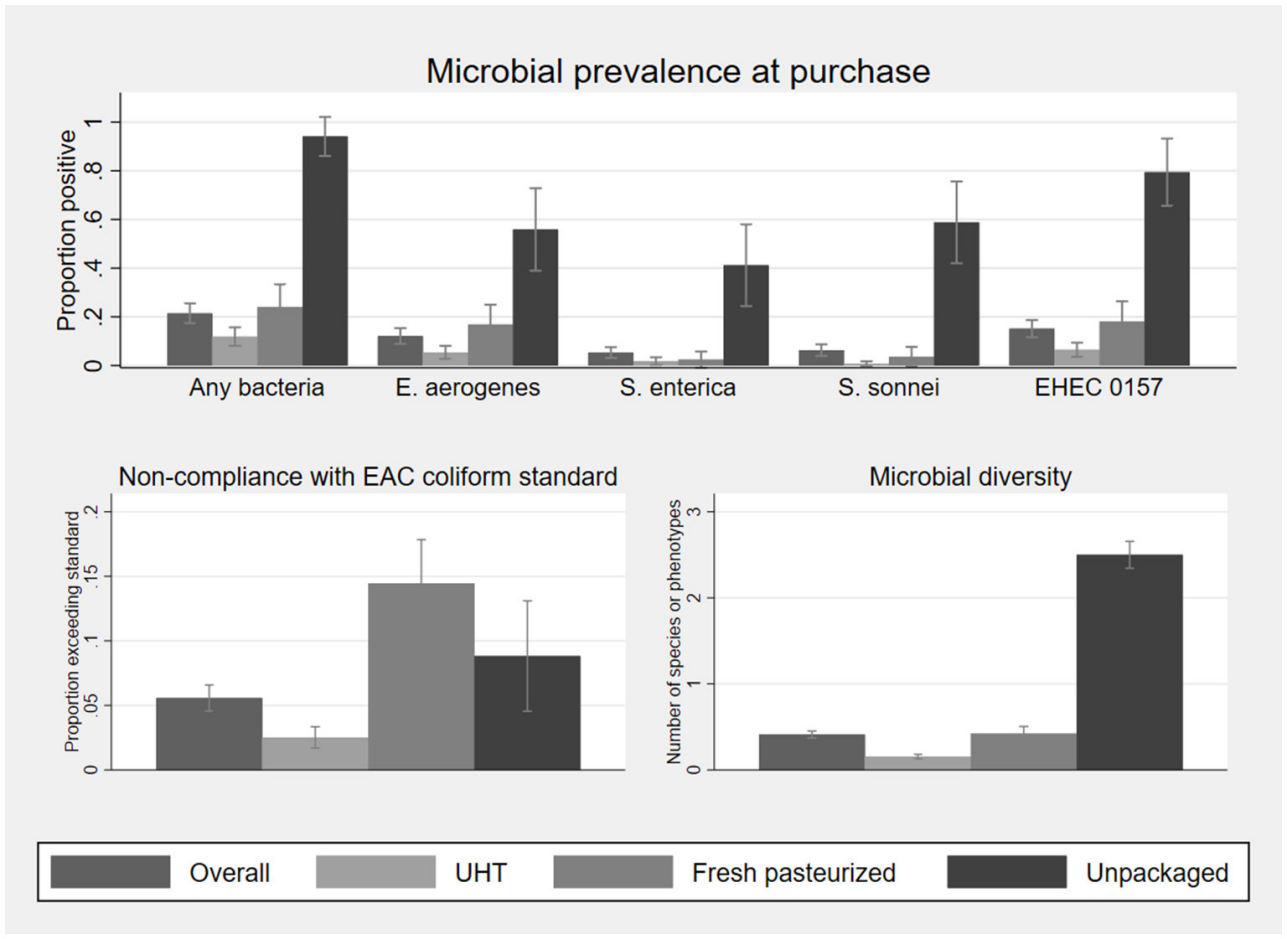
Microbial prevalence, compliance with EAC coliform standards for pasteurized (UHT, fresh pasteurized) and raw (unpackaged) milk, and microbial diversity, of 395 fluid milk samples collected at point of purchase, by milk type. Sample sizes by milk type are 278 UHT, 83 fresh pasteurized, and 34 unpackaged. Statistics on which this figure is based, as well as mean bacterial concentration among positive samples, are shown in Supplementary Table S5. EHEC 0157 was classified based on phenotype and not validated as a human pathogen. Error bars represent 95% confidence intervals.

Microbial prevalence and diversity in milk samples collected at point of purchase, as well as compliance with the EAC standard, are described in Figure 2. Statistics on which this figure is based, as well as mean microbial concentration among positive samples, are shown in Supplementary Table S5. At least one type of bacteria was cultured from 21% of the 395 samples of fluid milk. The mean number of bacterial species or phenotypes cultured per sample was 0.41. Probability and diversity of bacterial contamination were significantly associated with milk type, with the lowest risk of contamination in UHT milk (12% positive, mean number of bacteria detected 0.15, *n* = 278) and the highest in unpackaged milk (24.1%, *n* = 34). No bacteria were cultured from the single sample of infant formula.

*E. aerogenes* was the most commonly identified organism (12% of samples), and 5.6% of samples exceeded the EAC coliform standard of 10 cfu/ml in pasteurized milk or 50,000 cfu/ml in raw milk. Fresh packed (17%), UHT (5.4%), and unpackaged milk (56%) had significant rates of contamination with *E. aerogenes*. Fresh pasteurized milk was more likely to exceed the EAC coliform standard (based on *E. aerogenes* contamination alone) than UHT milk but less likely than unpackaged milk, noting that unpackaged milk has a higher EAC standard compliance threshold.

*Salmonella* spp. were cultured in 6.8% (*n* = 27/395) of fluid milk samples, 5.3% (*n* = 21/395) of which were human pathogen *S. enterica. S. enterica* was significantly more common in unpackaged milk (41%, *n* = 14/34) than either packaged fresh (2.4%, *n* = 2/83) or UHT milk (1.8%, *n* = 5/278). Among the 21 *S. enterica* positive samples, 13 had a concentration below 100 cfu/mL, two had between 100 and 1,000 cfu/mL, four had between 1,000 and 10,000 cfu/mL, and two were above 100,000 cfu/mL.

All *S. sonnei* were *ipaH* positive, indicative of a human pathogen. *S. sonnei* contamination was also most common in unpackaged milk at 59% of samples (*n* = 20/34), compared to 3.6% (*n* = 3/83) of fresh pasteurized milk and 0.7% of UHT milk (*n* = 2/278). Among the 25 *S. sonnei* positive samples, the concentration was below 100 cfu/mL for 10, between 100 and 1,000 cfu/mL for nine, between 1,000 and 10,000 cfu/mL for one, and above 100,000 cfu/mL for five.

The EHEC 0157 phenotype was found in 15% of milk samples at purchase. All presumptive EHEC 0157 colonies were negative for the *rdbE* gene and could not be validated as a pathogen health hazard. One fresh pasteurized milk sample was positive for *Proteus* spp. (26 cfu/ml) at point of purchase. None of the 396 vendor samples analyzed were positive for *E. coli*. Due to the lack of variability in *E. coli* and *Proteus* spp. these outcomes are omitted from the subsequent analysis aside from their inclusion among the organisms used to assess presence of any bacteria and bacterial diversity in infant food.

As differences in microbial concentration across milk types were not normally distributed, statistical tests were not applied to these. However, for most of the organisms analyzed the mean of log_10_-transformed cfu/ml is generally highest in unpackaged milk (Supplementary Figure S1). Adjusting the comparisons of microbial prevalence and diversity for vendor refrigeration practices and brand through a multivariate logistic regression model did not significantly affect the microbial prevalence or diversity (Supplementary Table S6), and differences across milk type are similar to those shown in Figure 2 and Supplementary Table S5.

Fifteen brands of UHT milk and nine brands of fresh pasteurized milk were purchased by caregivers. Among the seven UHT brands for which at least seven vendor samples were analyzed, differences in the rates of non-compliance with the EAC coliform standard were statistically significant (Table 1, *p* < 0.001). The number of observations per brand of fresh pasteurized milk was insufficient to allow statistical analysis. Further analysis of EAC standard compliance by brand is reported in the Supplementary Materials.

**Table 1:** Non-compliance by brand and milk type, EAC coliform standard (based on *E. aerogenes* cfu/ml).

	**Brand**						
	**1**	**2**	**3**	**4**	**5**	**6**	**7**
Proportion non-compliant UHT samples (*n*)	0.25 (8)	0.00 (9)	0.00 (7)	0.00 (12)	0.02 (147)	16.7 (12)	0.00 (63)
Proportion non-compliant fresh pasteurized samples (*n*)			0.13 (62)			0.00 (8)	

*The EAC standard for coliform is no more than 10 cfu/ml in pasteurized milk and no more than 50,000 cfu/ml in raw milk*.

### Contamination in Infant Food

**Figure 3:**
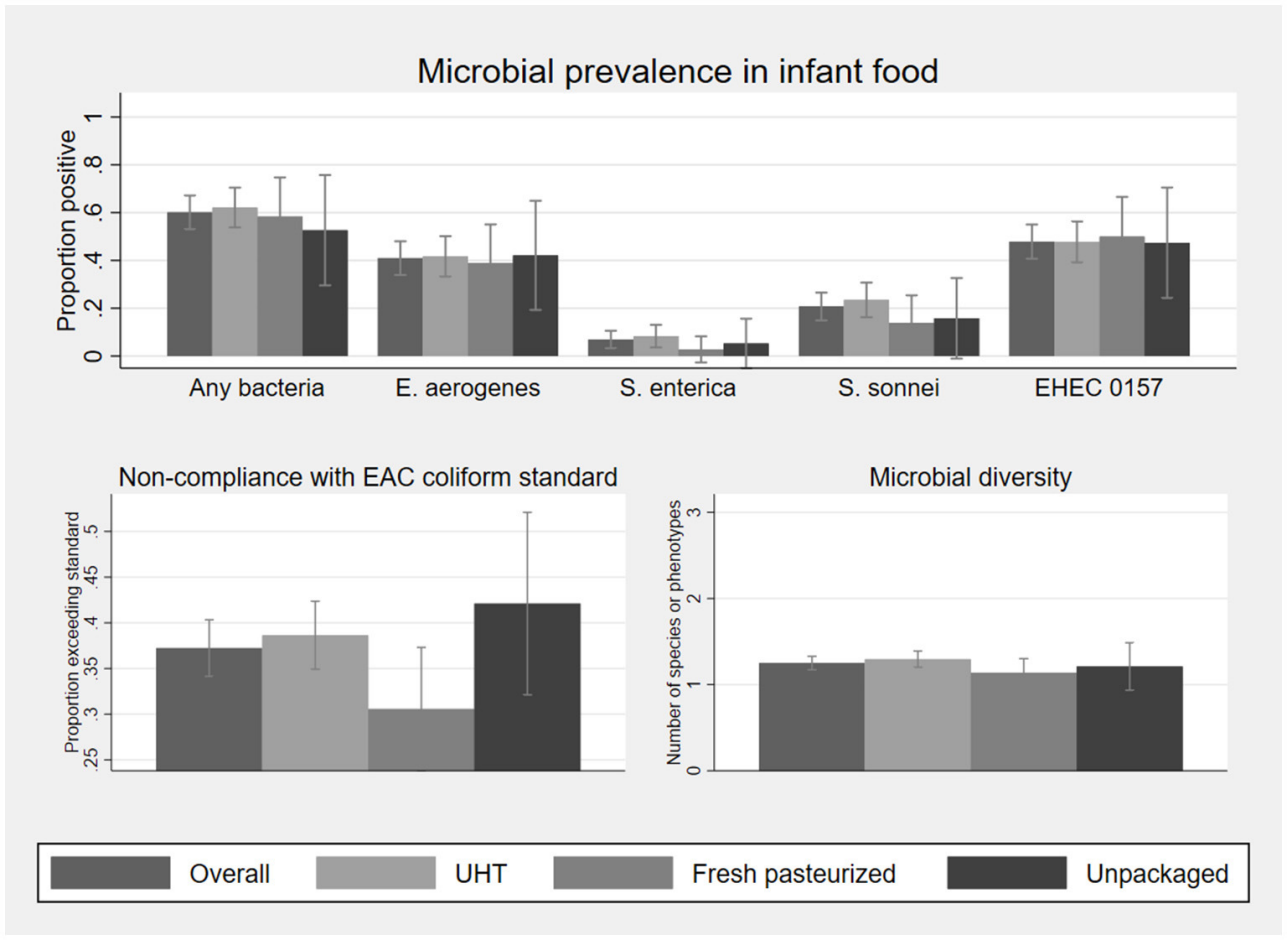
Microbial prevalence, compliance with EAC coliform standard for pasteurized milk, and microbial diversity, of 187 infant food samples matched to fluid milk samples collected at point of purchase, by milk type. Sample sizes by milk type are 132 UHT, 36 fresh pasteurized, and 19 unpackaged. Statistics on which this figure is based, as well as mean bacterial concentration among positive samples, are shown in Supplementary Table S5. EHEC 0157 was classified based on phenotype and not validated as a human pathogen. Error bars represent 95% confidence intervals.

After household handling and storage of infant food made with milk, contamination rates across milk types did not differ at the statistical threshold of *p* < 0.05 (Figure 3 and Supplementary Table S7). Pooling observations across all types of milk, bacteria were cultured from 60% of infant food samples, with a mean of 1.3 bacterial species per sample, and a mean total count of log_10_ 3.2 cfu/ml among samples with any contamination.

To rule out that contrasting findings of differences in contamination by milk type at purchase but no differences in the infant food prepared with this milk are due the smaller sample size available for the latter, we calculate minimum detectable differences by milk type based on the observed prevalence in infant food samples and point of purchase sample sizes. We find that for all comparisons, even if the sample size for infant food had been the same as that for milk at point of purchase, the observed differences would have been too small to detect statistically (Supplementary Table S3).

Both the proportion of infant foods in which any bacteria were detected (McNemar's exact test), and average bacterial diversity (negative binomial GLM allowing correlation within paired samples), are significantly higher than at point of purchase overall (*p* < 0.001). *E. aerogenes, S. enterica, S. sonnei*, and the EHEC 0157 phenotype were detected in 41%, 7%, 21%, and 48% of infant foods, respectively. *E. aerogenes* counts exceeded the EAC coliform standard for pasteurized milk for 37% of samples. Differences in prevalence of contamination between milk at point of purchase and infant food prepared with this milk were significant for all bacteria at *p* < 0.01, except for *S. enterica*.

Splitting the sample by milk type, the prevalences for all of the bacteria studied were higher in infant food prepared with UHT milk than at point of purchase (*p* = 0.039 for *S. enterica*; for all other organisms *p* < 0.001). Infant food prepared with fresh pasteurized milk was also more likely to contain at least one type of bacteria than at purchase (*p* = 0.035), though only one organism, the EHEC 0157 phenotype, was significantly more likely to be detected in infant food than in the paired purchase sample (*p* = 0.023). In contrast, detection of any bacteria was less likely in infant food prepared with unpackaged milk compared to the paired purchase sample (*p* = 0.004). The difference in bacterial prevalence between unpackaged milk at purchase and paired infant food samples is significant for *S. enterica* (*p* = 0.008), *S. sonnei* (*p* = 0.001), and the EHEC 0157 phenotype (*p* = 0.008), but not for *E. aerogenes* (p=0.388).

We also estimated adjusted models controlling for how milk was used in infant feeding (Supplementary Table S8). This adjustment accounts for mixed infant foods to potentially increase in contamination due to adding other contaminated food ingredients, or to decrease in contamination due to cooking. We found that, *S. enterica* was less likely to be detected in cooked foods prepared with milk (tea, porridge) compared to uncooked milk (pure milk or milk in cold cereal), holding the influence of milk type (UHT, pasteurized, unpackaged) constant. Results on the influence of milk type on contamination are similar to those shown in Figure 3 and Supplementary Table S4.

### Sources of Pathogens in Infant Food

Among 187 matched sample pairs of fluid milk at purchase and infant food from Safe Start control group caregivers, *E. aerogenes* was detected in 56% of the 27 infant foods prepared with milk that tested positive for *E. aerogenes* at point of purchase, *S. enterica* was detected in 14% of the 14 infant foods prepared with *S. enterica* positive milk, *S. sonnei* was detected in 25% of the 16 infant foods prepared with *S. sonnei* positive milk, and the EHEC 0157 phenotype was found in 61% of infant foods prepared with milk prepared with milk positive for the EHEC 0157 phenotype (Supplementary Table S9).

**Figure 4:**
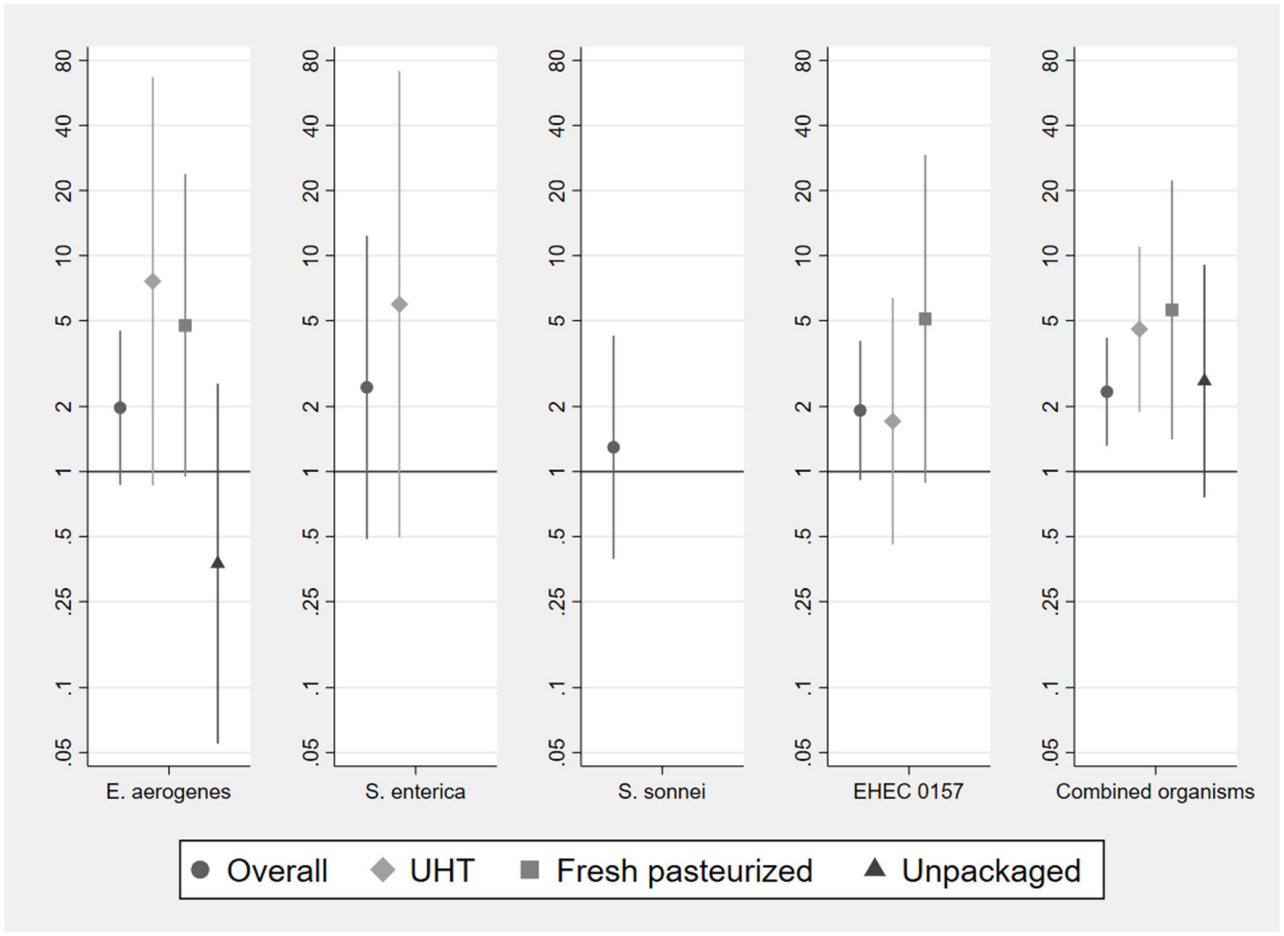
Odds ratios of bacterial detection in infant food based on vendor milk contamination status by type of milk used to prepare food. Samples sizes for analysis of individual organisms are 187 (overall, pooling milk types), 132 (UHT), 36 (fresh pasteurized), 19 (unpackaged). Sample sizes for analysis of combined organisms using stacked observations are 748 (overall), 528 (UHT), 144 (fresh pasteurized), 76 (unpackaged). Odds ratios for the presence of *S. enterica* and *S. sonnei* in fresh pasteurized and unpackaged milk, of *S. sonnei* in long-life milk, and for the EHEC 0157 phenotype in unpackaged milk, could not be estimated due to lack of variation in the outcome conditional on presence of organism in vendor sample. Error bars represent 95% confidence intervals.

Odds ratios of detection indicate that for any given bacterial species or phenotype, its presence in milk at purchase does not significantly predict its presence in infant food prepared with that milk (Figure 4). This holds when pooling the sample across milk types, and for the UHT, fresh pasteurized, and unpackaged samples separately. Point estimates of these odds ratios for UHT and fresh pasteurized milk are consistently above one, suggesting that this lack of significance may be driven limited statistical power.

Pooling the observations per microbial indicator, we have 748 observations in total (4 per paired sample of milk at point of purchase and infant food). We analyze odds ratios for these stacked observations, allowing the detection of different bacterial species or phenotypes to be correlated within paired samples (right-most panel of Figure 4, final panel of Supplementary Table S9). With this larger sample size, we find that the presence of a particular bacterial species or phenotype in any type of vendor milk is significantly associated with a greater risk of contamination with that same organism in infant food (OR = 2.34, 95% CI: 1.31, 4.17). This is the case for UHT (OR = 4.56, 95% CI 1.89, 10.99) and fresh pasteurized milk (OR = 5.60, 95% CI: 1.41, 22.3), but not for unpackaged milk (OR = 2.63, 95% CI: 0.76, 9.07). The probability of a bacterial species co-detection in infant food, conditional on detection in the milk used to prepare it at point of purchase, is significantly lower (*p* = 0.029) in unpackaged milk, at 0.33 (95% CI: 0.20, 0.47), than in UHT milk, at 0.65 (95% CI: 0.42, 0.88).

Changes in bacteria-specific contamination concentrations between vendor milk and infant food samples by processing and packaging status at purchase were examined to further understand the influence of household handling on bacterial transmission through milk. A large share of observations was negative for each species of bacteria in both milk at purchase and in infant food. The distribution of changes in concentration was thus not amenable to analysis as a continuous variable. We therefore discretized the change in concentration to a four-value categorical variable: not detected in either sample, lower concentration in infant food than in milk at point of purchase, higher concentration in infant food than milk at purchase, and maximum limit of detection in both samples. In only three cases were both the matched vendor milk and infant food sample contaminated at the maximum limit of detection. In all three cases, this occurred for the EHEC 0157 phenotype; one sample was UHT milk and two were unpackaged milk.

Table 2 shows the proportion of 187 matched samples (powder milk excluded) by fluid milk type and bacterial species or phenotype, in which microbial concentration was consistently zero, higher at purchase, and higher in the infant food. For all four types of bacteria, the probability that both matched samples tested negative is lowest in unpackaged milk. Among matched samples for which the level of concentration differed between point of purchase and infant food, food prepared with UHT milk was consistently more contaminated than the milk with which it was prepared. Infant food prepared with raw milk, in contrast, was consistently less contaminated relative to the milk used to prepare it at purchase. Fresh pasteurized milk generally followed the same pattern as UHT milk, with the exception of *S. enterica*, for which microbial concentration was higher in infant food than milk at point of purchase.

**Table 2:** Proportion of matched milk at point of purchase and infant food samples by categorical change in microbial contamination.

	**Overall**	**UHT**	**Fresh pasteurized**	**Un-packaged**	***p*-value^a^**
Sample size (all assays)	187	132	36	19	
***E. aerogenes***					
Not detected in either sample	0.53	0.58	0.53	0.16	0.000
95% CI	(0.45, 0.60)	(0.49, 0.66)	(0.36, 0.70)	(−0.02, 0.34)	
Lower in infant food than vendor milk	0.11	0.02	0.17	0.58	0.000
95% CI	(0.06, 0.15)	(0.00, 0.05)	(0.04, 0.29)	(0.33, 0.82)	
Higher in infant food than vendor milk	0.37	0.40	0.31	0.26	
95% CI	(0.30, 0.44)	(0.32, 0.49)	(0.15, 0.46)	(0.05, 0.48)	
***S. enterica***					
Not detected in either sample	0.87	0.90	0.92	0.53	0.000
95% CI	(0.82, 0.92)	(0.85, 0.95)	(0.82, 1.01)	(0.28, 0.77)	
Lower in infant food than vendor milk	0.06	0.02	0.06	0.42	0.001
95% CI	(0.03, 0.10)	(−0.01, 0.04)	(−0.02, 0.13)	(0.18, 0.67)	
Higher in infant food than vendor milk	0.07	0.08	0.03	0.05	
95% CI	(0.03, 0.11)	(0.04, 0.13)	(−0.03, 0.08)	(−0.06, 0.16)	
***S. sonnei***					
Not detected in either sample	0.73	0.77	0.81	0.32	0.000
95% CI	(0.66, 0.79)	(0.69, 0.84)	(0.67, 0.94)	(0.09, 0.55)	
Lower in infant food than vendor	0.07	0.00	0.06	0.63	0.000
95% CI	(0.04, 0.11)	(0.00, 0.00)	(−0.02, 0.13)	(0.39, 0.87)	
Higher in infant food than vendor milk	0.20	0.23	0.14	0.05	
95% CI	(0.14, 0.25)	(0.16, 0.31)	(0.02, 0.26)	(−0.06, 0.16)	
**EHEC 0157 phenotype** ^b^					
Not detected in either sample	0.45	0.49	0.44	0.11	0.000
95% CI	(0.38, 0.52)	(0.41, 0.58)	(0.27, 0.61)	(−0.05, 0.26)	
Lower in infant food than vendor	0.12	0.03	0.14	0.74	0.000
95% CI	(0.08, 0.17)	(0.00, 0.06)	(0.02, 0.26)	(0.52, 0.95)	
Higher in infant food than vendor milk	0.41	0.47	0.42	0.05	
95% CI	(0.34, 0.49)	(0.38, 0.56)	(0.25, 0.59)	(−0.06, 0.16)	

*The one infant formula sample is excluded*.

a *p-values refer to probability that the proportions for the respective rows or sets of rows (not detected in either sample; lower vs. higher in infant food than vendor milk) are equal across milk types, based on Fisher's exact test*.

b *Phenotype as indicated by culture analysis; the identity of these organisms could not be confirmed through PCR analysis*.

## Discussion

This study compared bacterial contamination patterns in infant foods and cow's milk used in their preparation to assess the role of the food system vs. household handling in contamination of infant food. We draw several notable conclusions from this study. First, a majority of the low-income urban caregivers who participated chose to purchase long-life milk, the most expensive type available, for their infants. We do not have data on milk consumed by other family members, and it is possible that the apparent consumer preference for pasteurized and UHT milk is specific to infant feeding. Representative data on milk consumption patterns in peri-urban Kisumu indicate that 60% of households regularly consume packaged milk, but do not specify which household members consume this milk, or distinguish between UHT and fresh pasteurized milk (25). Second, as generally expected, pasteurized packaged milk in this setting in Kenya was found to be microbiologically safer than unpackaged or raw milk. However, detection of enteric bacteria in both fresh pasteurized and UHT milk highlights problems with the formal dairy system. Of the 396 samples of milk obtained by caregivers of infants in Kisumu, 8.6% (34 samples) were found to be contaminated with *S. sonnei, S. enterica*, or both pathogens. While unpackaged milk samples were far more likely to be contaminated with one of these pathogens, pathogen contamination in UHT milk (2.2%) and fresh pasteurized milk (4.8%) were also non-trivial. Third, any benefits from purchasing UHT and fresh pasteurized milk disappeared by the time infants consumed these products. The frequency of overall and species-specific bacterial contamination in food fed to infants was similar for all milk types.

Examination of co-detection patterns in matched samples of milk at point of purchase and infant food across multiple individual bacterial species demonstrated that milk product safety issues and household hygiene both contributed to infant exposure to contaminated food. Microbial contamination of infant food most commonly arose during handling of food within the household, or from other food ingredients to which milk was added. This reinforces the importance of household-based interventions that can improve infant food preparation, feeding, and storage hygiene conditions. Yet, in multiple cases *S. sonnei* and *S. enterica* were detected in matched vended milk sources and infant foods. Pooling pathogenic and indicator organisms, the proportion of cases in which an organism present in vendor milk was also present after household handling and storage was highest in UHT milk, similar in fresh pasteurized milk, and lowest in unpackaged milk. Changes in microbial concentration between milk purchase and collection of infant food samples showed that infant food prepared with UHT milk was consistently more contaminated than the milk with which it was prepared, and infant food prepared with unpackaged milk was consistently less contaminated (if any change was observed).

These patterns suggest that how caregivers handle milk differs based on how it has been processed and whether it is purchased as a packaged or unpackaged product (9). The deterioration in microbial quality of infant food made with packaged milk could be due to a belief that packaged milk, especially that marketed as “Long Life” UHT milk, is safe for use without boiling and can be stored for a longer time after opening. This may be technically correct under the right conditions, but most households in this study lacked refrigeration for storing food, and kept food in containers with lids for periodic infant feeding throughout the day. Food storage and reuse allows for introduction of bacteria via the surface of the storage container, the hands of the caregiver or infant, or dust in the air, which combined with room temperature storage creates optimal conditions for bacterial growth.

The significance of the pathogens detected in food through this study in self-reported diarrhea and infection outcomes observed in the Safe Start trial remains to be assessed. Foodborne disease accounts for a significant level of diarrheal illness in children in Kenya (26). Data compiled by the Foodborne Disease Burden Epidemiology Reference Group of the WHO suggests that *S. enterica* and *Shigella* spp. are among the first and sixth most common causes of diarrheal foodborne illness globally (1, 26). While challenge studies among healthy adult volunteers in low burden, high-income countries report infectious doses of 10^5^ to 10^10^ organisms, data from foodborne gastroenteritis outbreaks in similar settings indicate that the infective dose of most wild *S. enterica* serovars may be as low as 10^1^ to 10^2^ organisms, regardless of high vs. low susceptibility (27, 28). The concentrations of *S. enterica* observed in milk samples collected at point of purchase in this study would be capable of causing disease if infants consume at least 25 mL of milk. Between 10 and 200 *S. sonnei* organisms can cause disease in healthy adults (29). Minimum thresholds for infection would have been met by consuming 25 mL of milk for 92% of the 23 samples in which *S. sonnei* was detected. For infants, even samples with the lowest level of contamination could be dangerous. The findings thus indicate cause for concern about the safety for infants of both packaged processed milk and unpackaged milk sold in Kisumu.

A limitation of this study is that our analysis of pathogen transmission from purchased milk to infant food was limited by relatively low prevalence of pathogens in vended milk sources. This may be due to testing of 2 mL or 2 gram volumes of food for processing. We also relied on a single non-selective pre-enrichment step for recovery of injured bacteria before plating on selective and differential agar, rather than on both primary and selective secondary enrichment steps. Preliminary spiking experiments with non-injured *Salmonella* spp. confirmed that this approach resulted in high recovery rates and accurate quantification of contamination at the point of sampling (manuscript pending). But, milk products and infant foods in Kenya likely contained a mixture of both healthy viable bacteria and heat-injured bacteria from pasteurization, and our protocols may have resulted in misclassification of some true positives as negatives. Bias from misclassification is most likely non-differential, meaning we may have underestimated the prevalence of food contamination, but conclusions of sub-group comparisons would be minimally affected. Additionally, we used cost-effective phenotype or gene-level typing methods to assess probable relatedness between milk at purchase and matched infant food samples. Determination of base pair relatedness via sequencing would have improved certainty in transmission conclusions but was beyond the scope of this project. The strength of our conclusions instead relies upon observing repeat patterns for a variety of bacteria species including common indicators and relatively rare pathogens. A shocking number of infant foods were contaminated by a bacteria phenotype matching EHEC 0157 (sorbitol negative, β-D-glucuronidase negative). EHEC 0157 identity was not validated by PCR screening and remains unidentified.

## Data Availability Statement

The raw data supporting the conclusions of this article will be made available by the authors, without undue reservation.

## Ethics Statement

Approval for the collection of infant food samples was obtained from Great Lakes University of Kisumu (Ref: GREC/010/248/2016), London School of Hygiene and Tropical Medicine (Ref: 14695), and the University of Iowa (Ref: 00000099). Written informed consent to participate in this study was provided by the participants' legal guardian/next of kin.

## Author Contributions

VH, KKB, and SS conceived of and designed the research study. JM, KT, and SS implemented the research. JM and OC contributed essential resources. VH and KKB analyzed the data and wrote the first draft. DKS provided statistical oversight. All authors contributed to the article and approved the submitted version.

## Funding

This study was supported by the CGIAR Research Program on Agriculture for Nutrition and Health (A4NH), hosted by IFPRI, and the Dutch Ministry of Foreign Affairs through SNV (Netherlands Development Organization) under the Voices for Change Partnership. The Safe Start trial, on which the study built, was funded by the United Kingdom Department for International Development (DFID) through the SHARE Research Consortium (ITDCHA2310).

## Conflict of Interest

The authors declare that the research was conducted in the absence of any commercial or financial relationships that could be construed as a potential conflict of interest.

## Publisher's Note

All claims expressed in this article are solely those of the authors and do not necessarily represent those of their affiliated organizations, or those of the publisher, the editors and the reviewers. Any product that may be evaluated in this article, or claim that may be made by its manufacturer, is not guaranteed or endorsed by the publisher.

## References

[1] WHO. WHO Estimates of the Global Burden of Foodborne Diseases. World Health Organization, 2007-2015. Fdberg (2015).

[2] Lanata CF. Studies of food hygiene and diarrhoeal disease. Int J Environ Health Res. (2003) 13(Suppl. 1):S175–83. 10.1080/0960312031000102921 12775394

[3] Kung'u JK Boor KJ Ame SM Ali NS Jackson AE Stoltzfus RJ. Bacterial populations in complementary foods and drinking-water in households with children aged 10-15 months in Zanzibar, Tanzania. J Health Popul Nutr. (2009) 27:41–52. 10.3329/jhpn.v27i1.3316 19248647 PMC2761806

[4] Black RE Brown KH Becker S Alim AR Merson MH. Contamination of weaning foods and transmission of enterotoxigenic *Escherichia coli* diarrhoea in children in rural Bangladesh. Trans R Soc Trop Med Hyg. (1982) 76:259–64. 10.1016/0035-9203(82)90292-9 7048652

[5] Barrell RA Rowland MG. Infant foods as a potential source of diarrhoeal illness in rural West Africa. Trans R Soc Trop Med Hyg. (1979) 73:85–90. 10.1016/0035-9203(79)90136-6 442188

[6] Agarwal DK Chandra S Bhatia BD Sanyal SC Agarwal KN. Bacteriology of weaning foods in some areas of Varanasi. Indian Pediatr. (1982) 19:131–4. 6749677

[7] Tsai K Simiyu S Mumma J Aseyo RE Cumming O Dreibelbis R. Enteric pathogen diversity in infant foods in low-income neighborhoods of Kisumu, Kenya. Int J Environ Res Public Health. (2019) 16:506. 10.3390/ijerph16030506 30759722 PMC6388216

[8] Kiambi S Onono JO Kang'ethe E Aboge GO Murungi MK Muinde P. Investigation of the governance structure of the Nairobi dairy value chain and its influence on food safety. Prev Vet Med. (2020) 179:105009. 10.1016/j.prevetmed.2020.105009 32438204

[9] Grace D Omore A Randolph T Kang'ethe E Nasinyama GW Mohammed HO. Risk assessment for *Escherichia coli* O157:H7 in marketed unpasteurized milk in selected East African countries. J Food Prot. (2008) 71:257–63. 10.4315/0362-028X-71.2.257 18326173

[10] Grace D Monda J Karanja N Randolph TF Kang'ethe EK. Participatory probabilistic assessment of the risk to human health associated with cryptosporidiosis from urban dairying in Dagoretti, Nairobi, Kenya. Trop Anim Health Prod. (2012) 44:33–40. 10.1007/s11250-012-0204-3 22886443

[11] Kenya National Bureau of Statistics. Kenya Integrated Household Budget Survey 2015-2016. Nairobi: Ministry of Devolution & National Planning (2016).

[12] Argwings-Kodhek G M'mboyi F Muyanga M Gamba P. Consumption Patterns of Dairy Products in Kenya's Urban Centres: Report from an Urban Household Survey Nairobi. Kenya: Tegemeo Institute of Agricultural Policy and Development (2005).

[13] Claeys WL Cardoen S Daube G De Block J Dewettinck K Dierick K. Raw or heated cow milk consumption: review of risks and benefits. Food Control. (2013) 31:251–62. 10.1016/j.foodcont.2012.09.035

[14] Oliver SP Jayarao BM Almeida RA. Foodborne pathogens in milk and the dairy farm environment: food safety and public health implications. Foodborne Pathog Dis. (2005) 2:115–29. 10.1089/fpd.2005.2.115 15992306

[15] United Nations Children's Fund (UNICEF). Estimates of Child Cause of Death, Diarrhoea. New York, NY. (2018). Available online at: https://data.unicef.org/wp-content/uploads/2018/02/CoD_Diarrhoea_Feb-2018_WHO_MCEE_234.xlsx (accessed December 29, 2021).

[16] Mumma J Simiyu S Aseyo E Anderson J Czerniewska A Allen E. The safe start trial to assess the effect of an infant hygiene intervention on enteric infections and diarrhoea in low-income informal neighbourhoods of Kisumu, Kenya: a study protocol for a cluster randomized controlled trial. BMC Infect Dis. (2019) 19:1066. 10.1186/s12879-019-4657-0 31856747 PMC6923833

[17] Mumma JAO Cumming O Simiyu S Czerniewska A Aseyo RE Muganda DN. Infant food hygiene and childcare practices in context: findings from an urban informal settlement in Kenya. Am J Trop Med Hyg. (2020). 102:220–222. 10.4269/ajtmh.19-0279 31746311 PMC6947802

[18] Aseyo RE Mumma J Scott K Nelima D Davis E Baker KK. Realities and experiences of community health volunteers as agents for behaviour change: evidence from an informal urban settlement in Kisumu, Kenya. Hum Resour Health. (2018) 16:53. 10.1186/s12960-018-0318-4 30286763 PMC6172748

[19] Simiyu S Czerniewska A Aseyo ER Baker KK Cumming O Odhiambo Mumma JA. Designing a food hygiene intervention in low-income, peri-urban context of Kisumu, Kenya: application of the trials of improved practices methodology. Am J Trop Med Hyg. (2020). 102:1116–1123. 10.4269/ajtmh.19-0629 32157996 PMC7204591

[20] Gibson AM Bratchell N Roberts TA. Predicting microbial growth: growth responses of salmonellae in a laboratory medium as affected by pH, sodium chloride and storage temperature. Int J Food Microbiol. (1988) 6:155–78. 10.1016/0168-1605(88)90051-7 3275296

[21] Mattick KL Phillips LE Jorgensen F Lappin-Scott HM Humphrey TJ. Filament formation by Salmonella spp. inoculated into liquid food matrices at refrigeration temperatures, and growth patterns when warmed. J Food Prot. (2003) 66:215–9. 10.4315/0362-028X-66.2.215 12597479

[22] Center for Food Safety and Applied Nutrition. Bacteriological Analytic Manual, 8th ed, Revision A. Silver Spring, MD: US Food and Drug Administration (1998).

[23] East African Community. EAST AFRICAN STANDARD: Pasteurized Milk — Specification. Arusha. (2006).

[24] East African Community. EAST AFRICAN STANDARD: Raw Cow Milk — Specification. Tanzania. (2006).

[25] Standards KBo. Kenya Standard 1552:2016: Code of Hygienic Practice for Milk and Milk Standards. Nairobi: Government of Kenya (2016).

[26] Hoffmann V Baral S. Foodborne Disease in Kenya: County-Level Cost Estimates and the Case for Greater Public Investment. Washington, DC: International Food Policy Research Institute Project Note, 1–21. (2019). 10.2499/p15738coll2.133525

[27] Teunis PF Kasuga F Fazil A Ogden ID Rotariu O Strachan NJ. Dose-response modeling of Salmonella using outbreak data. Int J Food Microbiol. (2010) 144:243–9. 10.1016/j.ijfoodmicro.2010.09.026 21036411

[28] Kothary MH Babu US. Infective dose of foodborne pathogens in volunteers: a review. J Food Safety. (2001) 21:49–68. 10.1111/j.1745-4565.2001.tb00307.x

[29] DuPont HL Levine MM Hornick RB Formal SB. Inoculum size in shigellosis and implications for expected mode of transmission. J Infect Dis. (1989) 159:1126–8. 10.1093/infdis/159.6.1126 2656880

