# Supplementary material for: Milk Product Safety and Household Food Hygiene Influence Bacterial Contamination of Infant Food in Peri-Urban Kenya
Source: Front Public Health. 2022 Feb 8;9:772892. doi: 10.3389/fpubh.2021.772892 (PMC8861079; doi:10.3389/fpubh.2021.772892)
Supplement: Supplementary file 1 [file Data_Sheet_1.docx]

**SUPPLEMENTAL MATERIALS**

**Cost of Milk**

The mean prices of the three main milk types purchased by study caregivers per 250 ml were 33 Kenyan Shillings (KSh) (CI 31, 35) (United States Dollar (USD): 0.30; CI 0.29, 0.32) for UHT, 28.1 KSh (CI 27, 29) (USD: 0.26; CI 0.24, 0.27) for fresh pasteurized, and 25 KSh (CI 22, 29) (USD: 0.23; CI 0.20, 0.26) for unpackaged milk.

**Statistical Power for characterization of contamination and comparisons by milk type**

We collected data on 395 fluid milk sources and 187 matched infant foods made with those sources, which is considered a moderate to large sample size for risk surveillance studies. Larger sample sizes would be useful for detecting rarer pathogen contamination patterns, such as in UHT and fresh packed milk, but these sample sizes were sufficient to demonstrate large differences in bacterial food contamination across milk products, and the lack of difference in contamination in infant food at the point of consumption. In the case of *S. enterica*, the pathogen with lowest prevalence in the study, the sample size of 278 for UHT milk purchases and detection rate of 1.8% imply that there is a 95% chance that the real rate of *S. enterica* contamination is within ±1.6% of the measured 1.8% value. The probability that the true *S. enterica* contamination rate in this type of milk is actually zero is less than the standard statistical threshold for Type I error of 5%. Further, chances that UHT milk has a similar contamination level as unpacked milk (for which the detection rate is 41.2%) is infinitely small. The 95% confidence interval for *S. enterica* detection in fresh packaged milk, at 2.4% ±3.3%, overlaps with that of UHT, but is again clearly distinct from the observed level in unpackaged milk. If we consider overall bacterial detection rate for purchased samples, there is a 95% chance that the real value for UHT milk (N=278) is within ±3.8% of the measured rate of 11.9%, a 9.2% margin of error (ME) around the 24.1% detection rate for fresh packed milk, and a 2.3% ME for the 9.4% detection rate for unpacked milk. All of these detection rates differ significantly from one another at the standard 95% confidence level typically used in statistical analysis.

  In Table S1, we show the minimum detectable differences between contamination rates by milk type for samples collected at point of purchase at 95% confidence and 80% power. Differences observed in our study data are greater than the minimum detectable differences for all comparisons between either type of packaged milk (UHT and Fresh Pasteurized) versus unpackaged milk. While the minimum detectable differences were smallest for comparisons between the two packaged milk types, observed differences were also smaller between these categories, resulting in relatively lower statistical power to distinguish between contamination levels in UHT versus fresh packaged milk of the magnitudes observed.

| **Table S1**: Minimum detectable difference in milk at point of purchase, by milk type and bacteria or phenotype, based on study sample sizes and observed contamination rate in first category, with power = 0.8 and alpha = 0.05 | | | |
| --- | --- | --- | --- |
|  | UHT vs Fresh pasteurized | UHT vs Unpackaged | Fresh pasteurized vs Unpackaged |
| Any bacteria | **0.130** | 0.192 | 0.268 |
| *E. aerogenes* | 0.101 | 0.150 | 0.250 |
| *S. enterica* | **0.065** | 0.098 | 0.160 |
| *S. sonnei* | **0.058** | 0.087 | 0.173 |
| EHEC-0157 phenotype | **0.141** | 0.207 | 0.254 |
| *Note:* Minimum detectable differences greater than actual differences are shown in bold. | | | |

We have fewer infant food samples, leading to larger margins of error of estimated bacterial detection rates relative to those for purchased milk, especially for food prepared with fresh pasteurized and unpackaged milk, for which the sample sizes are 36 and 19, respectively. However, we note that even for unpackaged milk, the probability that the true rate of contamination with any bacteria is zero can be soundly rejected, as the lower bound of the 95% confidence interval is 27.9%. Lower bounds of any bacterial contamination in infant foods prepared with UHT and fresh pasteurized milk are 53.7% and 41.4% respectively. We note that the confidence intervals for detection of particular pathogens do overlap with zero (*S. enterica* and *S. sonnei* for infant foods prepared with unpackaged milk; *S. enterica* for food prepared with fresh pasteurized milk). The moderate sample size for collected infant food samples, combined with these prevalence rates, limits our ability to test for differences in detection rates of specific pathogens by milk type.

In Table S2, we show the minimum detectable differences between contamination rates by milk type for infant food at 95% confidence and 80% power. Differences observed in our study data are less than the minimum detectable differences for all comparisons of infant food by type of packaged milk (UHT and Fresh Pasteurized) versus unpackaged milk. This indicates that we lack statistical power to detect the observed differences by milk type in the infant food samples, although it is not clear from this analysis whether the relative lack of power (compared to the vendor analysis) arises from the smaller differences in contamination rates observed in infant food or the smaller sample size.

| **Table S2**: Minimum detectable difference in infant food by milk type used to prepare it and bacteria or phenotype, based on study sample sizes and observed contamination rate in first category, with power = 0.8 and alpha = 0.05 | | | |
| --- | --- | --- | --- |
|  | UHT vs Fresh | UHT vs Unpackaged | Fresh vs Unpackaged |
| Any bacteria | **0.234** | **0.293** | **0.268** |
| *E. aerogenes* | **0.259** | **0.331** | **0.250** |
| *S. enterica* | **0.186** | **0.246** | **0.160** |
| *S. sonnei* | **0.243** | **0.316** | **0.173** |
| EHEC-0157 phenotype | **0.256** | **0.325** | **0.254** |
| Note: Minimum detectable differences greater than actual differences are shown in bold. | | | |

We therefore calculate minimum detectable effect differences by milk type based on the observed detection rates in infant food samples, combined with vendor milk sample sizes and show these in Table S3. We find that for all comparisons, even if the sample size for infant food had been the same as that for vendor milk, the observed differences would have been too small to detect at conventional levels of statistical precision (alpha = 0.05, power = 0.8). We therefore conclude that lack of statistical power is not the reason for our finding that contamination in infant food is not significantly influenced by the type of milk used to prepare it.

| **Table S3**: Minimum detectable difference in infant food by milk type used to prepare it and bacteria or phenotype, based on vendor sample sizes and observed contamination rate of infant food in first category, with power = 0.8 and alpha = 0.05 | | | |
| --- | --- | --- | --- |
|  | UHT vs Fresh | UHT vs Unpackaged | Fresh vs Unpackaged |
| Any bacteria | **0.162** | **0.228** | **0.259** |
| *E. aerogenes* | **0.174** | **0.249** | **0.281** |
| *S. enterica* | **0.116** | **0.172** | **0.165** |
| *S. sonnei* | **0.159** | **0.231** | **0.239** |
| EHEC-0157 phenotype | **0.173** | **0.247** | **0.273** |
| Note: Minimum detectable differences greater than actual differences are shown in bold. | | | |

**Additional results tables**

**Table S4**. Number of caregiver milk purchases by source and type, refrigeration and storage status

|  | UHT | Fresh Pasteurized | Un-packaged | Infant formula | Total |
| --- | --- | --- | --- | --- | --- |
| Duka | 277 | 82 | 0 | 1 | 360 (91%) |
| *Refrigerated* | *45* | *63* |  | *0* |  |
| Milk bar, of which | 0 | 0 | 21 | 0 | 21 (5.3%) |
| *Refrigerated* |  |  | *16* |  |  |
| *In wide-mouthed container with lid* |  |  | *16* |  |  |
| *In wide-mouthed container without lid* |  |  | *2* |  |  |
| *In containers for sale* |  |  | *1* |  |  |
| *In other container type* |  |  | *2* |  |  |
| Roadside vendor | 0 | 0 | 2 | 0 | 2 (0.51%) |
| *Refrigerated* |  |  | *0* |  |  |
| *In wide-mouthed container with lid* |  |  | *2* |  |  |
| Supermarket | 1 | 1 | 0 | 0 | 2 (0.51%) |
| *Refrigerated* | *1* | *0* |  |  |  |
| Neighbour | 0 | 0 | 7 | 0 | 7 (1.8%) |
| *Refrigerated* |  |  | *0* |  |  |
| *In wide-mouthed container with lid* |  |  | *2* |  |  |
| *In wide-mouthed container with no lid* |  |  | *4* |  |  |
| *In containers for sale* |  |  | *1* |  |  |
| Own cow | 0 | 0 | 4 | 0 | 4 (1.0%) |
| *Refrigerated* |  |  | *0* |  |  |
| *In wide-mouthed container with no lid* |  |  | *1* |  |  |
| *In containers for sale* |  |  | *3* |  |  |
| Combined sources | 278 | 83 | 34 | 1 | 396 (100%) |
| *Refrigerated* | *47* | *63* | *16* | *0* |  |
| *In wide-mouthed container with lid* |  |  | *20* |  |  |
| *In wide-mouthed container with no lid* |  |  | *7* |  |  |
| *In containers for sale* |  |  | *5* |  |  |
| *In other container type* |  |  | *2* |  |  |

*Note*: Full set of container types are listed only for combined milk sources; for specific sources, only containers observed at least once for that source are listed.

**Table S5.** Microbial detection rates, diversity, compliance with EAC coliform standard for milk, and mean concentrations in 395 fluid vendor milk samples at purchase, by milk treatment and packaging type

|  | Overall | UHT | Fresh Pasteurized | Unpackaged | p-value *^a^* |
| --- | --- | --- | --- | --- | --- |
| N (proportions, bacterial diversity) | 395 | 278 | 83 | 34 |  |
| Any bacteria |  |  |  |  |  |
| Proportion positive | 0.22 | 0.12 | 0.24 | 0.94 | 0.000 |
| 95% CI | [0.17, 0.26] | [0.08, 0.16] | [0.15, 0.34] | [0.86, 1.0] |  |
| Bacterial diversity | 0.41 | 0.15 | 0.42 | 2.5 | 0.000 |
| 95% CI | [0.32, 0.50] | [0.10, 0.21] | [0.23, 0.61] | [2.1, 2.9] |  |
| Mean log_10_ cfu/ml, if any | 2.9 | 1.8 | 2.1 | 4.5 |  |
| 95% CI | [2.5, 3.3] | [1.3, 2.3] | [1.5, 2.7] | [4.0, 5.0] |  |
| N | 85 | 33 | 20 | 32 |  |
| *E. aerogenes* |  |  |  |  |  |
| Proportion positive | 0.12 | 0.054 | 0.17 | 0.56 | 0.000 |
| 95% CI | [0.09, 0.15] | [0.027, 0.081] | [0.086, 0.25] | [0.38, 0.73] |  |
| Mean log_10_ cfu/ml, if any | 2.3 | 1.5 | 1.7 | 3.2 | 0.000 |
| 95% CI | [1.9, 2.7] | [0.82, 2.3] | [1.2, 2.3] | [2.7, 3.8] |  |
| N | 48 | 15 | 14 | 19 |  |
| > EAC standard ^b^ | 0.056 | 0.025 | 0.14 | 0.088 |  |
| 95% CI | [0.033, 0.078] | [0.007, 0.044] | [0.067, 0.22] | [-0.012, 0.19] |  |
| *S. enterica* |  |  |  |  |  |
| Proportion positive | 0.053 | 0.018 | 0.024 | 0.41 | 0.000 |
| 95% CI | [0.031, 0.075] | [0.002, 0.034] | [-0.010, 0.058] | [0.24, 0.59] |  |
| Mean log_10_ cfu/ml, if any | 2.2 | 1.1 | 1.8 | 2.7 | - |
| 95% CI | [1.6, 2.8] | [0.50, 1.7] | [1.7, 1.9] | [1.9, 3.5] |  |
| N | 21 | 5 | 2 | 14 |  |
| *S. sonnei* |  |  |  |  |  |
| Proportion positive | 0.063 | 0.007 | 0.036 | 0.59 | 0.000 |
| 95% CI | [0.039, 0.087] | [-0.003, 0.017] | [-0.005, 0.077] | [0.41, 0.76] |  |
| Mean log_10_ cfu/ml, if any | 2.5 | 0.95 | 0.62 | 2.9 |  |
| 95% CI | [1.8, 3.2] | [-3.5, 5.4] | [-0.75, 2.0] | [2.2, 3. 7] |  |
| N | 25 | 2 | 3 | 20 |  |
| EHEC-0157 phenotype *^c^* |  |  |  |  |  |
| Proportion positive | 0.15 | 0.065 | 0.18 | 0.79 | 0.000 |
| 95% CI | [0.12, 0.19] | [0.036, 0.094] | [0.096, 0.27] | [0.65, 0.94] |  |
| Mean log_10_ cfu/ml, if any | 3.1 | 2.3 | 2.0 | 4.3 |  |
| 95% CI | [2.7, 3.6] | [1.5, 3.1] | [1.3, 2.8] | [3.8, 4.8] |  |
| N | 60 | 18 | 15 | 27 |  |
| Notes: The one infant formula sample is excluded.  ^a^ P-values represent the probability that proportions or pathogen diversity are statistically identical across milk types, based on Fisher's exact test (for binary indicators) or a likelihood ratio test from a negative binomial regression (pathogen diversity). | | | | | |
| *^b^* Colony-forming units of *E*. aero*genes* are compared against the EAC standard for coliform contamination. The standard differs depending on whether milk is sold raw (50,000 cfu) versus processed (10 cfu).  *^c^* Phenotype as indicated by culture analysis; the identity of these organisms could not be confirmed through PCR analysis. | | | | | |

**Table S6**. Marginal effects of milk type and observed refrigeration on microbial presence and diversity in vendor milk.

|  | (1) | (2) | (3) | (4) | (5) | (6) | (7) |
| --- | --- | --- | --- | --- | --- | --- | --- |
|  | Any bacteria detected | Bacterial diversity | *E. aerogenes* detected | > EAC coliform standard | *S. enterica* detected | *S. sonnei* detected | EHEC-0157 phenotype detected |
| Milk type |  |  |  |  |  |  |  |
| UHT (Reference) | - | - | - | - | - | - | - |
| Fresh pasteurized | 0.220* | 0.300 | 0.098 | 0.088 | 0.042 | 0.076 | 0.210* |
|  | (0.119) | (5.841) | (0.108) | (0.090) | (0.045) | (0.071) | (0.120) |
| Unpackaged | 0.830*** | 0.768 | 0.381 | 0.994*** | 0.997*** | 0.999*** | 0.690*** |
|  | (0.061) | (19.681) | (0.268) | (0.004) | (0.001) | (0.000) | (0.204) |
| Modifier |  |  |  |  |  |  |  |
| Refrigerated | 0.089 | 0.020 | 0.039 | 0.018 | 0.004 | 0.020 | 0.091* |
|  | (0.068) | (0.328) | (0.039) | (0.021) | (0.011) | (0.019) | (0.048) |
|  |  |  |  |  |  |  |  |
| Observations | 387 | 394 | 387 | 378 | 306 | 186 | 364 |
| P(pasteurized=unpacked) | 0.005 | 0.479 | 0.283 | 0.000 | 0.000 | 0.000 | 0.102 |
| *Notes:* Marginal effects on the probability of detection, as estimated through logistic regression, are shown in columns 1, 3, 4, 5, 6, 7. Marginal effects on the number of organisms detected, as estimated through a negative binomial GLM, are shown in column 2. Standard errors of the marginal effects are shown in parentheses. Reference milk type is UHT. Binary brand indicators are included for brands represented by at least 7 observations. Observations are dropped when brand perfectly predicts failure to detect an organism, resulting in variation in the number of observations included across models. *** p<0.01, ** p<0.05, * p<0.1 | | | | | | | |
|  | | | | | | | |

**Table S7.** Microbial detection rates, diversity, compliance with EAC coliform standard for pasteurized milk, and concentration in 187 infant food samples matched to fluid vendor milk samples, by milk treatment and packaging type

|  | Overall | UHT | Fresh Pasteurized | Unpackaged | p-value *^a^* |
| --- | --- | --- | --- | --- | --- |
| N (proportions, bacterial diversity) | 187 | 132 | 36 | 19 |  |
| Any bacteria |  |  |  |  |  |
| Proportion positive | 0.60 | 0.62 | 0.58 | 0.53 | 0.446 |
| 95% CI | [0.53, 0.68] | [0.54, 0.71] | [0.41, 0.75] | [0.28, 0.77] |  |
| Bacterial diversity | 1.3 | 1.3 | 1.1 | 1.2 | 0.794 |
| 95% CI | [1.1, 1.4] | [1.1, 1.5] | [0.76, 1.5] | [0.54, 1.9] |  |
| log_10_ cfu / ml | 3.2 | 3.2 | 3.3 | 3.2 |  |
| 95% CI | [2.9, 3.5] | [2.9, 3.5] | [2.4, 4. 1] | [2.0, 4.4] |  |
| N | 113 | 82 | 21 | 10 |  |
| *E. aerogenes* |  |  |  |  |  |
| Proportion positive | 0.41 | 0.42 | 0.39 | 0.42 | 0.552 |
| 95% CI | [0.34, 0.48] | [0.33, 0.50] | [0.22, 0.56] | [0.18, 0.67] |  |
| log_10_ cfu / ml | 2.3 | 2.3 | 2.1 | 2.2 |  |
| 95% CI | [2.0, 2.5] | [2.0, 2.6] | [1.2, 3.0] | [1.7, 2.8] |  |
| N | 77 | 55 | 14 | 8 |  |
| > EAC standard *^b^* | 0.37 | 0.39 | 0.31 | 0.42 | 0.254 |
| 95% CI | [0.30, 0.44] | [0.30, 0.47] | [0.15, 0.46] | [0.18, 0.67] |  |
| *S. enterica* |  |  |  |  |  |
| Proportion positive | 0.070 | 0.083 | 0.028 | 0.053 | 0.642 |
| 95% CI | [0.033, 0.11] | [0.036, 0.13] | [-0.029, 0.084] | [-0.058, 0.16] |  |
| log_10_ cfu / ml | 1.6 | 1.7 | 1.3 | 1.8 |  |
| 95% CI | [1.3, 2.0] | [1.2, 2.1] | - | - |  |
| N | 13 | 11 | 1 | 1 |  |
| *S. sonnei* |  |  |  |  |  |
| Proportion positive | 0.21 | 0.23 | 0.14 | 0.16 | 0.428 |
| 95% CI | [0.15, 0.27] | [0.16, 0.31] | [0.020, 0.26] | [-0.023, 0.34] |  |
| log_10_ cfu / ml | 2.1 | 2.1 | 2.5 | 1.7 |  |
| 95% CI | [1.7, 2.6] | [1.6, 2.6] | [0.46, 4.5] | [-2.6, 6.0] |  |
| N | 39 | 31 | 5 | 3 |  |
| EHEC-0157 phenotype *^c^* |  |  |  |  |  |
| Proportion positive | 0.48 | 0.48 | 0.50 | 0.47 | 0.807 |
| 95% CI | [0.41, 0.55] | [0.39, 0.56] | [0.33, 0.67] | [0.23, 0.72] |  |
| log_10_ cfu / ml | 3.4 | 3.4 | 3.6 | 3.2 |  |
| 95% CI | [3.1, 3.7] | [3.0, 3.7] | [2.7, 4.4] | [1.8, 4.6] |  |
| N | 90 | 63 | 18 | 9 |  |

*Notes:* The one infant formula sample is excluded.

*^a^* P-values represent the probability that proportions or pathogen diversity are statistically identical across milk types, based on Fisher's exact test (for binary indicators) or a likelihood ratio test from a negative binomial regression (pathogen diversity).

*^b^* Colony-forming units of *E. aerogenes* are compared against the EAC standard for coliform contamination in pasteurized milk. The standard differs depending on whether milk is sold raw (50,000 cfu) versus processed (10 cfu).

*^c^* Phenotype as indicated by culture analysis; the identity of these organisms could not be confirmed through PCR analysis.

**Table S8**. Marginal effects of milk and food type on microbial presence and diversity in 187 infant food samples from Safe Start control households that used fluid milk to prepare infant food.

|  | (1) | (2) | (3) | (4) | (5) | (6) | (7) |
| --- | --- | --- | --- | --- | --- | --- | --- |
|  | Any bacteria detected | Bacterial diversity | *E. aerogenes* detected | > EAC coliform standard | *S. enterica* detected | *S. sonnei* detected | EHEC-0157 phenotype detected |
| Milk type ^a^ |  |  |  |  |  |  |  |
| UHT (Reference) | - | - | - | - | - | - | - |
| Fresh pasteurized | -0.029 | -0.147 | -0.031 | -0.082 | -0.049* | -0.089 | 0.027 |
|  | (0.092) | (0.299) | (0.094) | (0.091) | (0.028) | (0.067) | (0.094) |
| Unpackaged | -0.106 | -0.097 | 0.019 | 0.049 | -0.031 | -0.077 | -0.013 |
|  | (0.124) | (0.383) | (0.121) | (0.119) | (0.035) | (0.082) | (0.125) |
| Food type ^b^ |  |  |  |  |  |  |  |
| Milk and milk-cereal (Reference) | - | - | - | - | - | - | - |
| Porridge | -0.050 | -0.097 | 0.082 | 0.082 | -0.088** | -0.045 | -0.052 |
|  | (0.075) | (0.258) | (0.077) | (0.076) | (0.036) | (0.062) | (0.077) |
| Tea | 0.065 | -0.010 | 0.069 | 0.103 |  | 0.008 | -0.003 |
|  | (0.135) | (0.441) | (0.139) | (0.140) |  | (0.108) | (0.137) |
|  |  |  |  |  |  |  |  |
| Observations | 187 | 187 | 187 | 187 | 171 ^c^ | 187 | 187 |
| p (fresh pasteurized =unpackaged) | 0.591 | 0.914 | 0.727 | 0.347 | 0.754 | 0.926 | 0.781 |
| *Notes:* Marginal effects on the probability of detection, as estimated through logistic regression, are shown in columns 1, 3, 4, 5, 6, 7. Marginal effects on the number of organisms detected, as estimated through a negative binomial GLM, are shown in column 2. Standard errors of the marginal effects are shown in parentheses.  ^a^ The estimated marginal effects for other milk types (fresh pasteurized, unpackaged) represent differences in probability of detection or bacterial diversity compared to UHT milk, holding constant whether the milk was provided alone or with cold cereal, or as an ingredient in tea or porridge.  ^b^ The estimated marginal effects for other food types (porridge, tea) represent differences in probability of detection or bacterial diversity compared to this category, holding milk type (UHT, fresh pasteurized, unpackaged) constant.  ^c^ Food type = tea predicts absence of *S. enterica* perfectly; 16 observations dropped. | | | | | | | |

**Table S9.** Odds ratio (95% CI) of bacterial detection in infant food based on milk contamination status at purchase by milk treatment and packaging type, and sub-group conditional probabilities (95% CI) that organism is detected in infant food sample

|  | Overall | UHT | Fresh Pasteurized | Unpackaged |  |
| --- | --- | --- | --- | --- | --- |
| *E. aerogenes* | 2.0  [0.87, 4.5] | 7.6  [0.86, 67] | 4.8  [0.95, 24] | 0.38  [0.055, 2.6] |  |
| N | 187 | 132 | 36 | 19 |  |
| P of detection if: |  |  |  |  | p-value ^a^ |
| Positive at purchase | 0.56  [0.36, 0.76] | 0.83 *^b^* | 0.67 *^b^* | 0.33  [0.020, 0.65] | 0.113 |
| N | 27 | 6 | 9 | 12 |  |
| Negative at purchase | 0.39  [0.31, 0.46] | 0.40  [0.31, 0.48] | 0.30  [0.11, 0.48] | 0.57 *^b^* | 0.362 |
| N | 160 | 126 | 27 | 7 |  |
| *S. enterica ^c^* | 2.5  [0.49, 12] | 6.0  [0.50, 71] | - | - |  |
| N | 187 | 132 |  |  |  |
| P of detection if: |  |  |  |  | p-value |
| Positive at purchase | 0.14  [-0.066, 0.35] | 0.33 *^b^* | 0.00 *^b^* | 0.11 *^b^* | 0.604 |
| N | 14 | 3 | 2 | 9 |  |
| Negative at purchase | 0.064  [0.027, 0.10] | 0.078  [0.031, 0.12] | 0.029  [-0.030, 0.089] | 0.00  [0.00, 0.00] | 0.612 |
| N | 173 | 129 | 34 | 10 |  |
| *S. sonnei ^c^* | 1.3  [0.39, 4.3] | - | - | - |  |
|  | 187 |  |  |  |  |
| P of detection if: |  |  |  |  | p-value |
| Positive at purchase | 0.25  [0.012, 0.49] | 1.00 *^b^* | 0.00 *^b^* | 0.23  [-0.034, 0.50] | 0.293 |
| N | 16 | 1 | 2 | 13 |  |
| Negative at purchase | 0.20  [0.14, 0.27] | 0.23  [0.16, 0.30] | 0.15  [0.022, 0.27] | 0.00 *^b^* | 0.367 |
| N | 171 | 131 | 34 | 6 |  |
| EHEC-0157 phenotype *^d^* | 1.9  [0.91, 4.0] | 1.7  [0.46, 6.4] | 5.1  [0.89, 29] | - |  |
| N | 187 | 132 | 36 | 19 |  |
| P of detection if: |  |  |  |  | p-value |
| Positive at purchase | 0.61  [0.44, 0.78] | 0.60  [0.23, 0.97] | 0.78 *^b^* | 0.53  [0.26, 0.79] | 0.511 |
| N | 36 | 10 | 9 | 17 |  |
| Negative at purchase | 0.45  [0.37, 0.53] | 0.47  [0.38, 0.56] | 0.41  [0.21, 0.61] | 0.00 *^b^* | 0.545 |
| N | 151 | 122 | 27 | 2 |  |
| Combined organisms | 2.3  [1.3, 4.8] | 4.6  [1.9, 11] | 5.6  [1.4, 22] | 2.6  [0.76, 9.1] |  |
| N | 748 | 528 | 144 | 76 |  |
| P of detection if: |  |  |  |  | p-value |
| Positive at purchase | 0.46  [0.36, 0.57] | 0.65  [0.42, 0.88] | 0.59  [0.37, 0.81] | 0.33  [0.20, 0.47] | 0.075 |
| N | 93 | 20 | 22 | 51 |  |
| Negative at purchase | 0.27  [0.23, 0.30] | 0.29  [0.25, 0.33] | 0.20  [0.13, 0.28] | 0.16  [0.0055, 0.31] | 0.159 |
| N | 655 | 508 | 122 | 25 |  |

*Notes:* The one infant formula sample is excluded. The overall significance of the binomial GLM model used to test for differences in conditional probabilities across milk types, in which indicators for both unpackaged milk and fresh pasteurized milk are included, is 0.075.

*^a^* P-values for individual organisms represent the probability that the conditional probability of detection is statistically identical across milk types, based on Fisher's exact test. For combined organism types, p-value reflects a joint test of significance of milk type indicators in a binomial GLM model with standard errors clustered within paired samples.

*^b^* Confidence intervals are shown only for estimates based on at least 10 observations.

*^c^* For presence of *S. enterica* and *S. sonnei* in fresh pasteurized and unpackaged milk, of *S. sonnei* in long-life milk, and for the EHEC-0157 phenotype in unpackaged milk, odds ratios cannot be estimated due to lack of variation in the outcome conditional on presence of organism in vendor sample.

*^d^* Phenotype as indicated by culture analysis; the identity of these organisms could not be confirmed through PCR analysis.

**Microbial Concentration Distributions**

**Figure S1.** Histograms of microbial concentration in positive samples, by bacterial species and phenotype, milk treatment and packaging type, and point of sampling

**
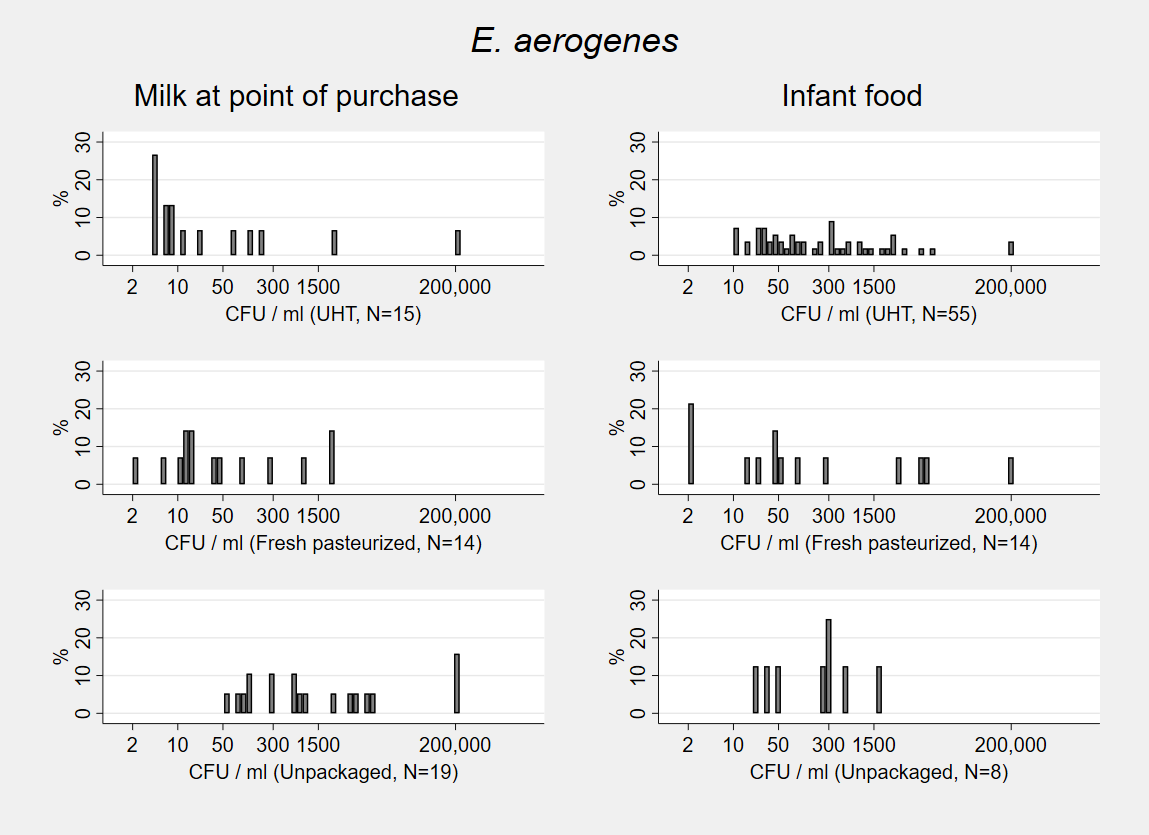
**

*
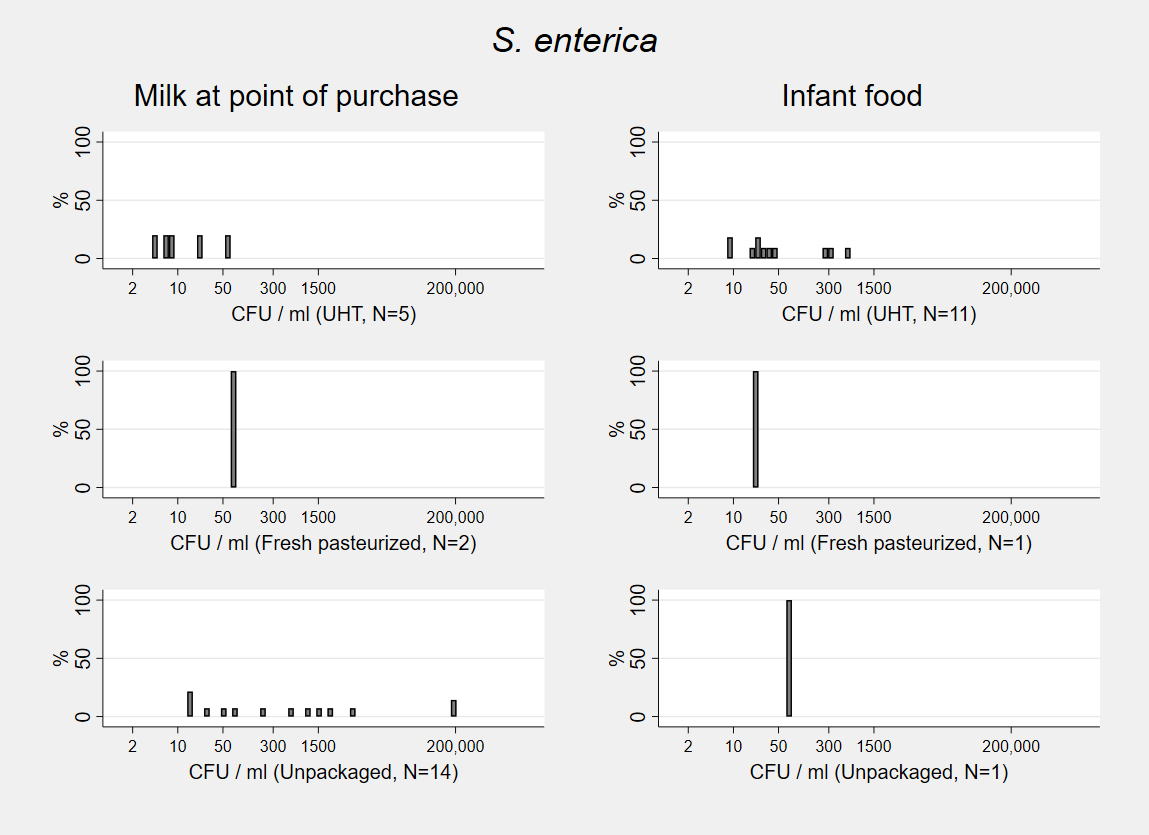
*

*
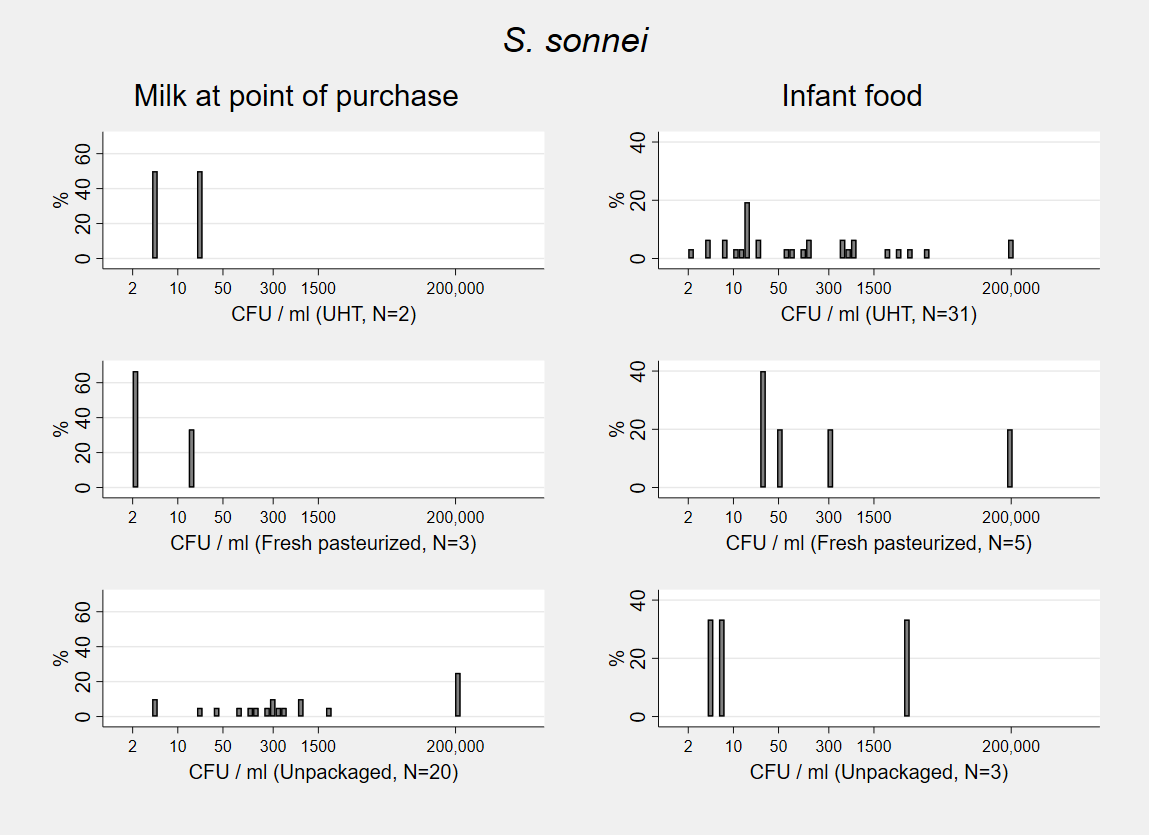
*

*
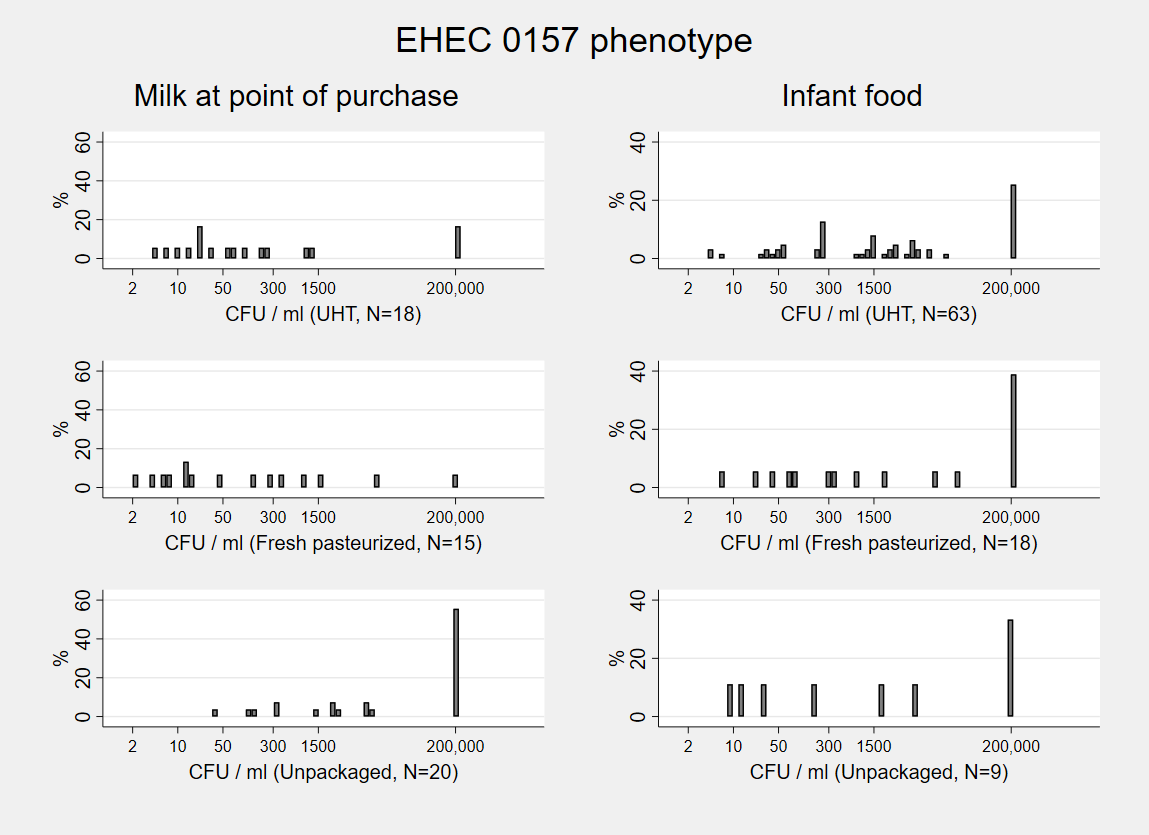
*

*Notes*: Based on analysis of 278 long life, 83 fresh pasteurized, and 34 unpackaged vendor samples; infant food samples from Safe Start control households only using long-life milk (132), fresh pasteurized milk (36), and unpackaged milk (19).

**Variability in microbial contamination by brand**

Sixteen different brands of milk were purchased by caregivers, and among these, eight included both UHT and fresh pasteurized milk. *E. aerogenes, S. enterica,* and *S. sonnei* were detected at some level in 62.5%, 31%, and 25% of brands, respectively. Among the seven UHT brands with at least seven observations (Table 2), those with the most observations (N=147 and N=63) had among the lowest rates (2% and 0%) of non-compliance while one of the brands with the fewest observations (N=8) had the highest rate of non-compliance (25%). A Fisher’s exact test on the rates of noncompliance with the EAC coliform standard based on *E. aerogenes* plate counts indicated that differences in the rate of non-compliance across brands were statistically significant (p<0.001). While the number of samples positive for pathogens were too few to conduct meaningful statistical analysis, six long-life milk samples contained *S. enterica* or *S. sonnei*. Contamination with either of the pathogens analyzed and the *E. aerogenes* compliance status of fresh milk, whether packaged or unpackaged, was not associated with refrigeration status.
